# Cytotoxic effects of nanosilver are highly dependent on the chloride concentration and the presence of organic compounds in the cell culture media

**DOI:** 10.1186/s12951-016-0244-3

**Published:** 2017-01-06

**Authors:** Jean-Pierre Kaiser, Matthias Roesslein, Liliane Diener, Adrian Wichser, Bernd Nowack, Peter Wick

**Affiliations:** 1 Particles-Biology Interactions Laboratory, Empa, Swiss Federal Laboratories for Materials Science and Technology, Lerchenfeldstrasse 5, 9014 St. Gallen, Switzerland; 2 Technology and Society Laboratory, Empa, Swiss Federal Laboratories for Materials Science and Technology, Lerchenfeldstrasse 5, 9014 St. Gallen, Switzerland

**Keywords:** CaCo-2 cells, Nanosilver, Culture conditions, Chloride concentration, Protein content, Cytotoxicity

## Abstract

**Background:**

Nanosilver shows great promise for use in industrial, consumer or medical products because of its antimicrobial properties. However, the underlying mechanisms of the effects of silver nanoparticles on human cells are still controversial. Therefore, in the present study the influence of the chloride concentration and different serum content of culture media on the cytotoxic effects of nanosilver was systematically evaluated.

**Results:**

Our results show that nanosilver toxicity was strongly affected by the composition of the culture media. The chloride concentration, as well as the carbon content affected the silver agglomeration and the complex formation. But also the dissolution of nanosilver and the availability of free silver ions (Ag^+^) were severely affected by the compositions of the culture media. Cells, only exposed to silver particles in suspension and dissolved silver complexes, did not show any effects under all conditions. Nanosilver agglomerates and silver complexes were not very soluble. Thus, cells growing on the bottom of the culture dishes were exposed to sedimented nanosilver agglomerates and precipitated silver complexes. Locally, the concentration of silver on the cell surface was very high, much higher compared the silver concentration in the bulk solution. The cytotoxic effects of nanosilver are therefore a combination of precipitated silver complexes and organic silver compounds rather than free silver ions.

**Conclusions:**

Silver coatings are used in health care products due to their bacteriostatic or antibacterial properties. The assessment of the toxicity of a certain compound is mostly done using in vitro assays. Therefore, cytotoxicity studies of nanosilver using human cell cultures have to be undertaken under well controlled and understood cultivations conditions in order to improve the compatibility of different studies. Especially when eukaryotic versus prokaryotic systems are compared for the evaluation of the use of nanosilver as antibacterial coatings for implants in order to prevent bacterial colonization.

**Electronic supplementary material:**

The online version of this article (doi:10.1186/s12951-016-0244-3) contains supplementary material, which is available to authorized users.

## Background

Nanosilver has unique physical and chemical features, and excellent antimicrobial properties. A variety of products containing nanoscale silver particles has been commercially available for over 100 years [1], including electronics, textiles, and medical products [2]. Because of this widespread use, there is an increasing potential of exposure and therefore the number of toxicity studies has increased greatly in the past years, but despite all the research it is still unclear whether nanosilver’s toxicity is related to its small size [3–5], or to the release of silver ions [6, 7], or a combination of these [8]. It has been reported that silver ions predominate in the toxicity [9, 10], and that oxidizing conditions are a prerequisite for any toxicity to occur [11]. Other studies described nanosilver of larger particle size (>10 nm) as apparently causing cytotoxic effects in, for example, macrophages or HepG2 cells [10, 12]. According to Singh et al. [13] nanosilver is internalized by cells and, once internalized, silver ions are released and these may interact with cellular compounds such as nucleic acids, sulfhydryl groups of metabolic enzymes or sulfur-containing cell components [14, 15].

Comprehensive nanomaterial characterization and the precise composition of the environment in which the biological effects were assessed must be known prior to studying the underlying mechanisms of nanosilver toxicity. In vitro assays have been performed in different cell culture media and various cell types have been used. The variety of protocols and media compositions is huge and the media vary in sodium chloride concentrations as well as protein content, which is mainly determined by the fetal calf serum (FCS) content, ranging from 0 to 10% or more. Many of the published studies investigating nanosilver toxicity have used submerged cell culture monolayers. Silver nanoparticles transferred into a cell culture medium will most likely agglomerate and therefore significantly change their diffusion and sedimentation behavior in the cell culture-well [16–18]. Independent of agglomeration, uncoated silver nanoparticles dissolve and release silver ions, which are highly reactive and bind strongly to electron donor groups and are thereby transformed into silver complexes with different bioavailability and toxicity [15, 18, 19].

The first evidence that the chemical composition of the environment has a significant influence on silver nanoparticle transformation and fate was when Handy et al. reported in 2008 that agglomeration behavior, ion release and transformation into other silver complexes differed in seawater compared with freshwater [17]. It is now known that salinity and the presence of organic matter have a major effect on the agglomeration, dissociation and solubility of nanoparticles, all relevant factors in the estimation of the ecotoxicological potential of nanosilver and its derivates [17, 20, 21]. After administration of nanosilver particles into a cell culture medium, they can sediment as particles or as agglomerates. In the presence of an oxidizing agent, such as oxygen or H_2_O_2_, silver ions are released from the nanoparticles and silver nanoparticle dissolution strongly increases under conditions of increased chloride concentration [22]. The released free silver ions will form silver chloride complexes in the presence of chloride, and these are sparingly soluble at low chloride concentrations and highly soluble at high chloride concentrations. The dominant equilibrium silver species shifts to the negatively charged silver chloride species (AgCl_x_^(x−1)^) [22]. The amount of free ionic silver in a physiological culture medium is therefore very low [23]. In addition, in the presence of proteins the surface of silver nanoparticles will be coated by a protein corona, which will significantly reduce the dissolution of nanoparticles [24, 25]. It can be further assumed that silver ions are complexed by proteins and inorganic compounds in the cell culture medium, and that nanosilver will be passivated in the presence of sulfur-containing compounds [24].

To our knowledge, there has not been any systematic and comprehensive experimental investigation providing evidence-based data concerning the influence of inorganic and organic compounds on the cytotoxicity of nanosilver. Therefore, in the present study the aim was to systematically evaluate the influence of the chloride concentration and different FCS content of culture media on the cytotoxic effects of nanosilver using human epithelial colorectal adenocarcinoma CaCo-2 cells cultures as a proof of concept.

The CaCo-2 cell line as a very simplified model of the gastrointestinal tract was chosen because food and food packaging may contain or release silver nanoparticles or silver ions and is broadly used and enables good benchmarking of our data with published data. An applied single concentration of 20 µg/mL silver nanoparticles was evaluated because the effect of that concentration on the viability of CaCo-2 cell cultures has been published previously [26]. A known concentration is prerequisite for this mechanistic study to evaluate the biological effect shifts applying to different cell culture conditions. In addition to cell viability, cell morphology, production of reactive oxygen species (ROS) and cytokine release were also assessed in the cell culture media with varying concentrations of chloride and FCS.

## Methods

### Nanosilver characterization

Nanosilver was obtained from PPG Industries Europe BV (The Netherlands). Its characterization was performed using different physicochemical methods, such as dynamic light scattering, zeta potential analysis and transmission electron microscopy (TEM). The characterization data have been published by Kaiser et al. [26].

The presence of endotoxin was analyzed with a kinetic chromogenic limulus amebocyte lysate assay (Charles Rivers Laboratories, Sulzfeld, Germany) as described by Smulders et al. [27].

The agglomeration behavior of nanosilver during incubation in each culture medium was analyzed by a particle tracking method using a NanoSight LM20 instrument (Malvern Instruments Ltd, UK, software version 2.3.5.0033.7-Beta7). The nanosilver particles (100 µg/mL) were incubated in culture media with 10% FCS at 37 °C for 0, 24, 48 and 72 h. The reliability of the size measurements with the NanoSight methodology was verified by an interlaboratory comparative round-robin test [28].

### Silver speciation

The chemical equilibrium model, Visual Minteq software version 3.0 was used to calculate the concentrations of free silver ions and other inorganic silver complexes in the culture media with different chloride concentrations [29]. The Visual Minteq software is suitable for samples with a known composition. The culture media contained high amounts of organic compounds, which formed organic complexes with ionic silver but whose complexation with silver is unknown.

The concentrations of all the main cations and anions in the culture media were known. For the organic compounds, the silver complexation of glutamine is known and was included in the calculation. For the calculation the pH was set to 7.4 and the temperature to 37 °C.

The complexation of silver in the culture media containing the organic supplements and FCS at concentrations of 1, 5 and 10% was estimated using an ion-selective electrode, (detection limit: 10 ng/mL, type Ag^+^/S2-ISE, Metrohm, Herisau, CH). The ISE was calibrated by chloride titration of a 10 ng/mL silver ion concentration with 0.5–50 mM chloride. The detection limit was 10 ng/mL. A 3-(N-morpholino) propanesulfonic acid buffer (0.1 M, pH 7.4) amended with NaNO_3_ (1 mM) was used for these chloride titrations. The free silver ion concentration in the calibration solution was calculated by Visual Minteq. Calibration curves were linear (R^2^ > 0.999) down to 1.75 e-10 M free silver ions.

Furthermore, we analyzed the total amounts of dissolved silver complexes in the different culture media. For this purpose the media were amended with nanosilver (20 µg/mL), respectively ionic silver (1.5 µg/mL), and incubated at 37 °C for 48 h. The total silver concentrations in samples taken from the upper sections of the culture media were measured by ICP-MS (Element 2 ICP-MS, Thermo/Finnigan, Reinach, CH). The detection limit of the instrument for silver was 0.3 ng/mL.

In addition, the amounts of silver ions and inorganic and organic silver complexes of low molecular weight (<3 kDa) were analyzed. The different culture media were inoculated with nanosilver (20 µg/mL), respectively ionic silver (1.5 µg/mL), and incubated at 37 °C for 48 h. Next, all the compounds in the media with higher molecular weight (>3 kDa) were removed by cut-off filters (3 kDa). The cut-off filters were centrifuged at 4000*g* for 30 min and the amount of silver in the filtrates was estimated by ICP-MS measurement.

### Cell cultures

CaCo-2 cells were obtained from Health Protection Agency Culture Collections (Salisbury, UK: order no. 86010202). The genotype (STR profiles according to ATCC documentation; CaCo-2 HTB-37) had been analyzed at the start of this study.

The cells were cultured in modified minimum essential Eagle medium (MEM) according to Sigma (product no. M2279, Buchs, CH), with different concentrations of heat-inactivated FCS (1, 5 and 10%) (Lonza, Verviers, B; Cat. No. DE14-801FH, Lot No. 9SBO22H2), 1% penicillin–streptomycin–neomycin-solution (PSN, Gibco, Life Technologies, Basel, CH), 1% glutamine solution (Gibco, Life Technologies), 1% non-essential amino acid solution (Sigma), 1 mM sodium pyruvate (Gibco, Life Technologies) and 1% vitamin solution (Sigma) under uniform cell culture conditions (5% CO_2_, 95% humidity and 37 °C). The cultures were passaged once weekly before use in the experiments.

### Culture media for the cytotoxicity assays

The cytotoxicity of nanosilver was investigated in 15 different culture media: 5 different chloride concentrations and 3 different FCS concentrations (1, 5 and 10%). Medium A corresponded to the original MEM (Sigma), with a chloride concentration of 124.5 mM. In culture media B–E the chloride concentration was reduced stepwise by 25% and replaced with sulfate (Table 1). Medium E contained a very low chloride concentration (0.05 mM).

**Table 1:** Cation and anion concentrations (mM) in modified minimum essential Eagle media

Cations and anions	Media A (mM)	Media B (mM)	Media C (mM)	Media D (mM)	Media E (mM)
Ca^+^	1.4	1.4	1.4	1.4	1.4
K^+^	5.4	5.4	5.4	5.4	5.4
Mg^+^	0.8	0.8	0.8	0.8	0.8
H^+^	28.2	28.2	28.2	28.2	28.2
Na^+^	143.6	143.6	143.6	143.6	143.6
Cl^−^	124.5	87.3	58.2	29.1	0.05
CO_3_^2−^	26.2	26.2	26.2	26.2	26.2
P (PO_4_^3−^)	1.0	1.0	1.0	1.0	1.0
S (SO_4_^2−^)	0.8	19.4	34.0	48.5	63.1

The culture media were supplemented with: fetal calf serum (1, 5, and 10%) (Lonza, Verviers, B; Cat. No. DE14-801FH, Lot No. 9SBO22H2), glucose (1 g/L), penicillin–streptomycin–neomycin solution (1%), glutamine solution (1%), non-essential amino acids solution (1%), sodium pyruvate (1%), MEM vitamin solution (1%), and phenol red (0.011 g/L).

The composition of culture medium A corresponded to the original culture medium MEM from Sigma. This original culture medium contained 124.5 mM sodium chloride. In the other culture media (media B–E) the sodium chloride concentrations were stepwise reduced by 25%. The culture medium E contained finally a minimal amount of 0.05 mM sodium chloride. The concentrations of the other anions and cations corresponded to the original Sigma MEM medium. The lower sodium chloride concentrations in the culture media B–E compared to culture medium A would lead automatically to a decrease of the ionic strength. In order to keep the ionic strength of all culture media similar, the stepwise reduction of the sodium chloride concentrations in culture media B–E was compensated with sodium sulfate. Sodium sulfate has no negative effects on cell viability as shown in Additional file 1: Figure S3.

### Floating cell cultures

CaCo-2 cells were precultivated for 3 days on coverslips in the original culture medium (Medium A). The coverslips with the adherent CaCo-2 cells were then placed, cells on the underside, onto the surface of each of the 15 different culture media, which had been supplemented with 20 µg/mL nanosilver. A spacer ensured that the coverslips were kept on the surface of the culture medium.

### Cell viability

Apoptosis/necrosis in cell cultures was quantitatively investigated by flow cytometric analysis based on the binding of annexin V fluorescein to phosphatidyl serine and incorporation of propidium iodide to distinguish between apoptotic and late apoptotic/necrotic cells. The staining procedure was performed according to the manufacturer’s protocol (PF032 Calbiochem, Germany) of annexin V binding with adherent cells. For the different chromophores the following excitation and emission wavelengths were used: annexin V-FITC (ex. 488 nm; em. 528 nm) and propidium iodide (ex. 488 nm; em. 635 nm). A total of 10,000 cells were counted in each flow cytometric analysis. Cadmium chloride (25 µM) was used as the positive control.

### Cell morphology

Morphology was analyzed by bright field microscopy using a phase contrast method at ×100 (Nikon-Diaphot, Egg, CH) after exposure of the CaCo-2 cells to 20 µg/mL nanosilver for 48 h in the different culture media.

### Nanoparticle uptake analysis in vitro

The TEM analysis of incorporated particles was performed with a Zeiss 900 TEM (Carl Zeiss MicroImaging, Jena, Germany), described in detail by Thurnherr et al. [30]. In brief: the cells were pelleted and sucked up into a capillary tube (Leica, Heerbrugg, CH). Fixation of the cells was performed with a 0.2 M sodium cacodylate buffer containing 3% glutaraldehyde. Fixed cells were postfixed in 2% osmium tetroxide in 0.1 M sodium cacodylate buffer for 30 min, dehydrated through a graded ethanol series followed by acetone and embedded in Epon resin (Fluka, Buchs, CH). Ultrathin sections were contrasted with 2% uranyl acetate and lead citrate before observation by TEM at 80 kV. The engulfed silver agglomerates in the cells were analyzed by scanning transmission electron microscopy and energy dispersive X-ray spectrometry (STEM/EDX).

### Release of reactive oxygen species

The release of ROS was estimated with the reactive dye 2′,7′-dichlorodihydrofluorescein-diacetate (H_2_DCF-DA), as described by Kaiser et al. [31]. The release of ROS was analyzed after exposure of the CaCo-2 cells for 1, 2, 3 and 4 h to 20 µg/mL nanosilver in salt solutions that corresponded to the chloride ion concentrations in culture media A–E. The salt solutions contained only the cations and anions of the corresponding culture media without FCS, phenol red, glutamine, PSN, glucose, non-essential amino acid solution, sodium pyruvate, and vitamin solution. 3-Morpholinosydnonimine hydrochloride (1 mM) was used as the positive control.

### Release of cytokines

CaCo-2 cells exposed to nanosilver released interleukin 8 (IL-8) into the culture media and the concentration of IL-8 was analyzed with an ELISA cytokine assay (IL-8 ELISA Ready-SET-Go, eBioscience, Frankfurt, Germany) according to the manufacturer’s protocol. Exposure of cells to tumor necrosis factor alpha (TNF-α, 50 ng/mL) for 24 h to induce the release of IL-8 was used in positive control cultures.

### Statistical analysis

The experiments were repeated independently three times. Significant effects were determined using Student’s *t* test. Differences were considered significant at p < 0.05. In the Figures, data are presented as the mean of three independent experiments and the standard error of the mean (SEM) over the mean experimental values of each of the three independent experiments.

## Results

### Agglomeration of nanosilver

Changes in the agglomeration of nanosilver in the media with different chloride concentrations (media A–E) were analyzed after incubating the particles in culture media with 10% FCS over 72 h. As soon as the silver nanoparticles came in contact with the culture media, agglomerates formed in all the media, independent of the chloride concentration. The size distribution of nanosilver was similar for all the different media and in the range of approximately 65–235 nm, with maxima approximately 150 nm (Additional file 1: Table S1). In the cell free culture media the silver agglomerates shifted within the first 24 h incubation period towards larger particles. After this 24 h incubation period the silver agglomerates didn’t increased anymore.

Some of the agglomerated particles precipitated and were adsorbed onto CaCo-2 cells growing on the bottom of the culture dishes (Additional file 1: Figure S1). At higher silver concentrations it could be demonstrated that the precipitated silver agglomerates were adsorbed by cells and formed micro-sized agglomerates on the plasma membrane of the cells (Additional file 1: Figure S2).

### Determination of dissolved silver in the upper sections of the culture media

**Fig. 1:**
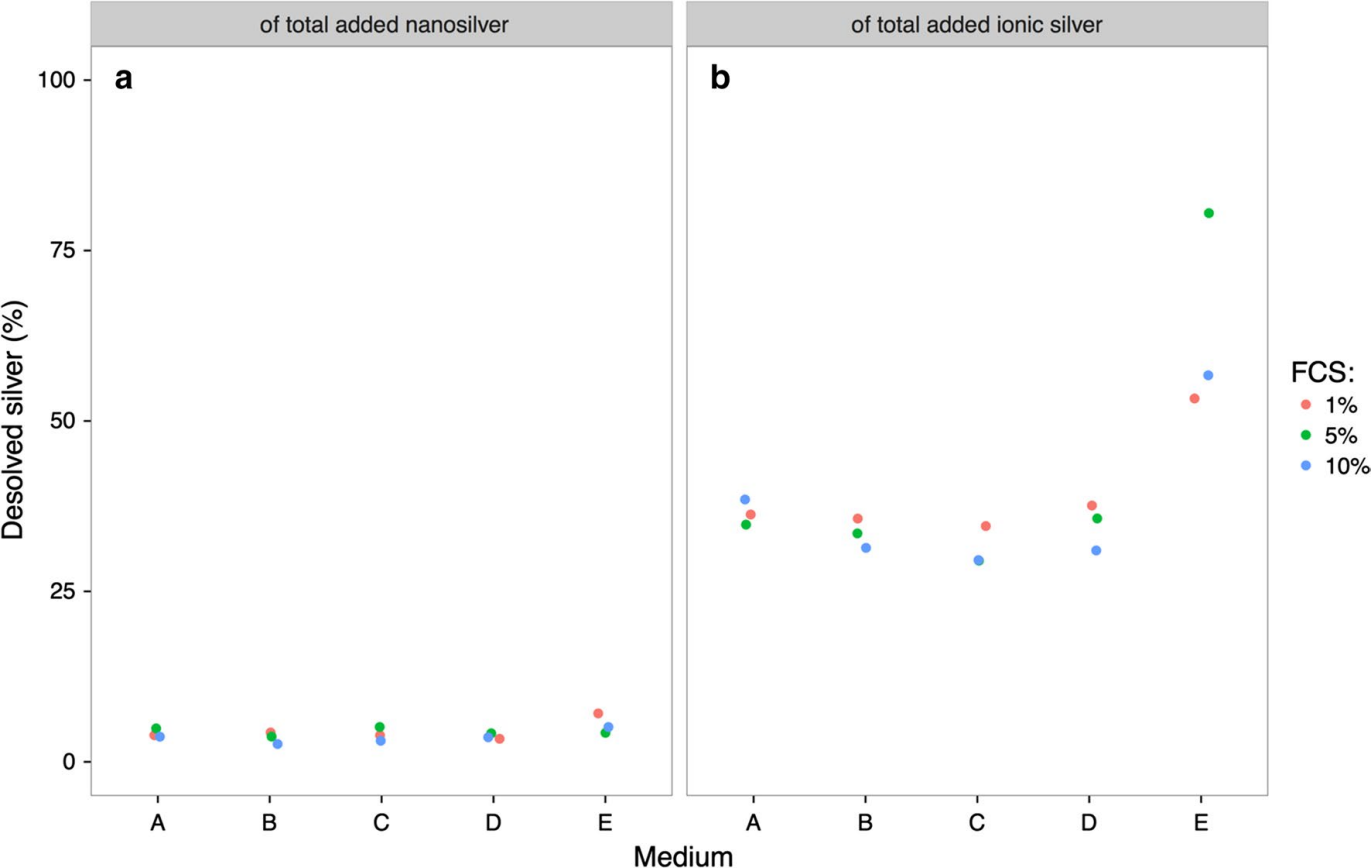
Measurements of total silver in the upper sections of culture media *A*–*E*. The samples were inoculated with 20 µg/mL nanosilver (**a**) or 1.5 µg/mL ionic silver (**b**) and incubated at 37 °C for 48 h

Nanosilver and ionic silver were inoculated into the different culture media for 48 h. The analysis of the upper sections of culture media A–D showed that 95–97% (19.0–19.5 µg/mL) of the added nanosilver (20 µg/mL) was removed from the upper section of the media within 48 h, due to sedimentation (Fig. 1a). When the same amount of nanosilver was added to culture medium E, less of the supplemented nanosilver was removed and 5–7.5% (1.0–1.5 µg/mL) of the applied nanosilver could be detected in the upper section of the culture medium. The amount of FCS did not influence the stability of the added silver (Fig. 1a).

When ionic silver was applied to the different culture media, similar behavior was observed. In culture media A–D, 30–40% (0.45–0.60 µg/mL) of the added ionic silver (1.5 µg/mL) was stable in solution (Fig. 1b). When the same amount of silver ions was added to culture medium E, the amount of silver remaining in solution was higher, 50–80% (0.8–1.2 µg/mL) of the applied silver could be detected in the upper section of culture medium E. The amount of FCS in the culture media did not affect the stability of the ionic silver, as already observed with the nanosilver particles.

### Determination of dissolved silver and silver complexes of low molecular weight (<3 kDa) in the culture media

**Fig. 2:**
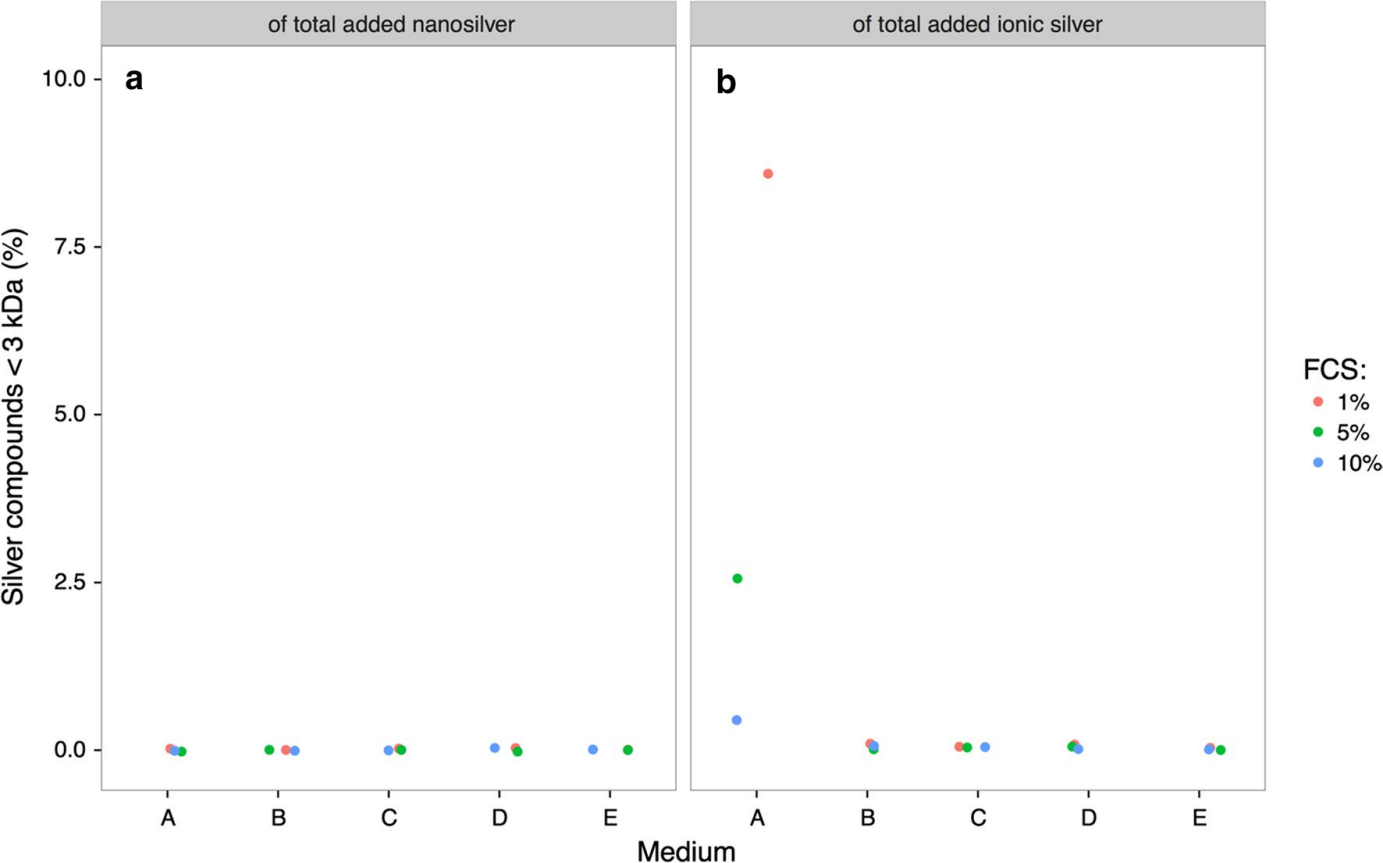
ICP-MS measurement of dissolved silver (<3 kDa) in the different culture media. The samples were inoculated with 20 µg/mL nanosilver (**a**) or 1.5 µg/mL ionic silver (**b**) and incubated at 37 °C for 48 h. Most of the released silver ions from nanosilver particles, as well as the applied ionic silver formed complexes with the proteins of the culture medium. Next, molecules with molecular weight higher than 3 kDa were removed by cut-off filters and the filtrates of the different culture media, which contained only low molecular weight molecules (<3 kDa), were analyzed by ICP-MS measurement

Dissolved silver (<3 kDa) in the different culture media was investigated with ICP-MS analysis after filtration. Nanosilver (20 µg/mL) and ionic silver (1.5 µg/mL) were inoculated into the different culture media and 48 h later all inorganic and organic particles of higher molecular weight (>3 kDa) were removed by ultrafiltration with cut-off filters (3 kDa). After the application of 20 µg/mL nanosilver to medium A with 1% FCS, 2.3 ng/mL silver could be measured in the filtrate. The silver concentrations in the media filtrates were dependent on the chloride and FCS concentrations of the culture media. In culture media B–E, which contained less chloride than culture medium A, the measured silver concentration in the filtrates was in the range of the detection limit (0.3 ng/mL) (Fig. 2a). When silver was applied as ionic silver (1.5 µg/mL) to culture medium A, between 130 and 6.4 ng/mL silver could be detected in the filtrate (Fig. 2b). The silver concentration was again dependent on the FCS concentration. Less silver was detected in the filtrates of culture media with higher FCS concentrations. Culture media B–E contained lower chloride concentrations than culture medium A. The measured amounts of silver in the media-filtrates were very low.

### Measurement of free silver ions in the culture media with ISE

The concentration of free silver ions in culture media A–D containing the organic supplements and FCS in the concentrations of 1, 5 and 10% was measured with the ISE. Immediately after adding the 5 µg/mL Ag^+^ to the media, the free silver ion concentration was 1.6e-10 M, 1.8e-10 M and 1.3e-10 M for medium A with 1, 5 and 10% FCS. For medium D the respective values were 8.6e-10 M, 5.9e-10 M and 3.4e-10 M. This means that only about 3e-4% of the total silver was present as free silver ions in medium A and 1e-3% for medium D. After 3 h the ISE measurement was below the calibration range, indicating a further complexation of the added silver.

### Calculation of the amounts of free silver and inorganic silver complexes

Speciation of silver was performed using Visual Minteq, including all major cations and anions and glutamate. For the additional organic compounds added to the medium no silver complexation constants are known, nor for FCS. Calculations were therefore performed with the known stability constants and considering the solubility of solid AgCl. For 1.5 µg/mL total silver, the predicted precipitation of solid AgCl in medium A was 28%, the remaining speciation of silver complexes was 0.08% Ag^+^, 7.6% AgCl_aq_, 80.7% AgCl_2_^−^ and 11.6% AgCl_3_^2−^. In medium E no AgCl was expected to precipitate and the Ag-speciation was 65.7% Ag^+^, 3.4% AgCl_aq_, 10.0% Ag-glutamate and 20.8% Ag_2_SO_4_. The speciation in the fraction smaller than 3 kDa after addition of nanosilver particles was also calculated. For medium A the species distribution was 0.08% Ag^+^, 7.7% AgCl_aq_, 80.7% AgCl_2_^−^ and 11.5% AgCl_3_^2−^. For medium E the speciation was 66.1% Ag^+^, 2.6% AgCl_aq_, 10.1% Ag-glutamate and 21% Ag_2_SO_4_. Medium A therefore predominately comprised dissolved silver-chloride species, whereas medium E was predominately free ionic silver and organic silver complexes. The amounts of ionic silver and dissolved silver complexes were very low in all culture media, and were unable to affect cell behavior when present in such low concentrations.

### Cytotoxicity assays

Control cultures were grown in the different culture media with different FCS concentrations. Annexin V/propidium iodide staining showed that the number of living cells remained comparable in all culture media A–E (Additional file 1: Figure S3).

**Fig. 3:**
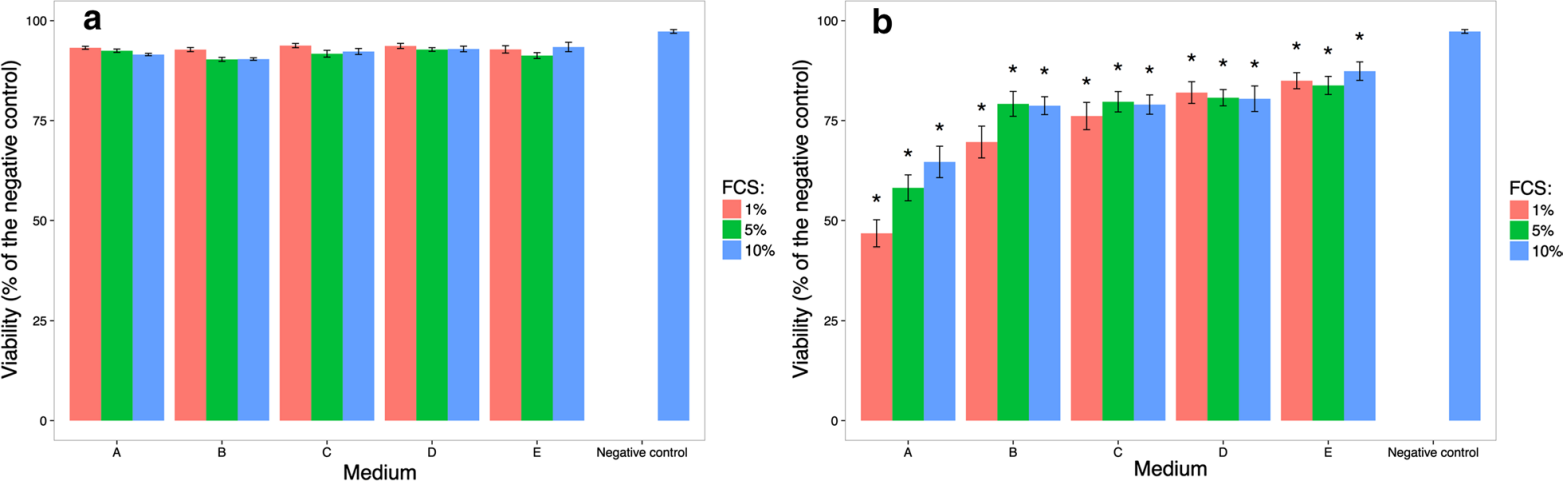
Viability of cell cultures grown in culture media with 20 µg/mL nanosilver. Floating cell cultures (**a**) and bottom cell cultures (**b**) were cultivated in media with different chloride concentrations (media *A*–*E*) in the presence of nanosilver for 48 h. The negative control culture corresponded to medium A with 10% fetal calf serum (FCS). *Asterisk* significantly different from the negative control

In order to elucidate the cytotoxicity of the silver remaining in the upper sections of the culture media, CaCo-2 cells were pre-cultivated on coverslips, each of which were placed with the adherent CaCo-2 cells facing the culture media, onto the surface of the 15 different culture media that had been inoculated with 20 µg/mL nanosilver. The chloride and FCS concentrations in the culture media did not noticeably influence the viability of the floating cell cultures (Fig. 3a). In contrast, cell cultures growing on the bottom of the culture dishes were heavily exposed to precipitating silver complexes and sedimented silver agglomerates (Fig. 3b). When these cell cultures were cultivated in medium A, which corresponded to the original MEM culture medium, in the presence of 20 µg/mL nanosilver, a higher number of dead cells was observed (Fig. 3b). Thus the chloride concentration in the culture medium increased the cytotoxic effect of the applied nanosilver. Increasing the FCS concentration of the culture medium increased the amount of living cells, independent of the presence of chloride or sulfate.

**Fig. 4:**
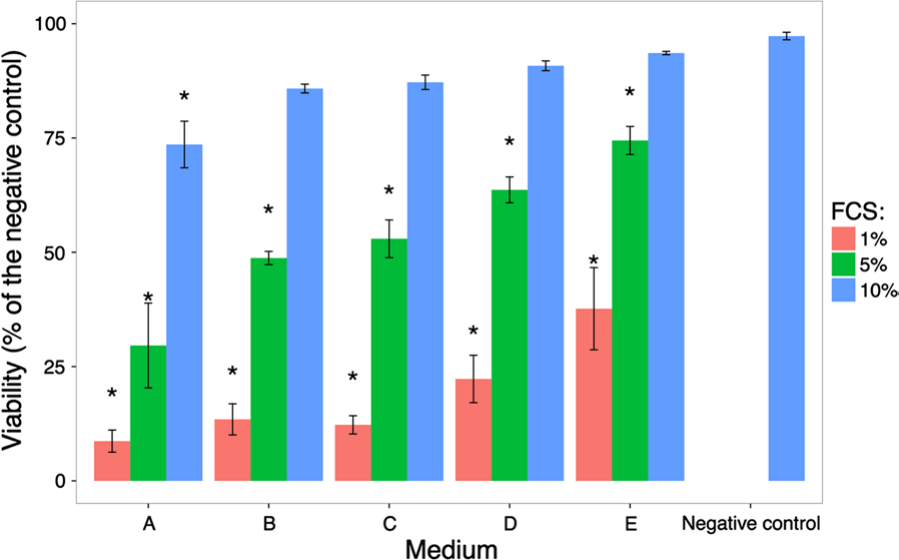
Viability of cell cultures grown in culture media with 1.5 µg/mL silver ions. Cells were cultivated in culture media with different chloride concentrations (media *A*–*E*) in the presence of 1.5 µg/mL ionic silver for 48 h. The negative control culture corresponded to medium A with 10% fetal calf serum (FCS). *Asterisk* significantly different from the negative control

The same investigations were done using ionic silver instead of nanosilver. The viability of cell cultures growing on the bottom of the culture dishes was far less affected by ionic silver (1.5 µg/mL) when the cells were growing in a medium with lower chloride concentrations (e.g. media B–E) and with higher FCS content (Fig. 4).

Nanosilver also affected the morphology of the CaCo-2 cells growing on the bottom of the culture dishes. The effects were dependent on the chloride concentration of the culture medium. The morphology of cell clusters was mostly affected when the cells were growing in medium A (Additional file 1: Figure S4A). Cell growth decreased and the cells formed smaller clusters. Cells growing on the periphery of the clusters were especially affected. No dead cells could be observed in the center of the cell clusters. However, the CaCo-2 cells were much less affected when growing in media with lower chloride, respectively higher sulfate, concentrations (media B–E). The floating cell cultures were not exposed to the precipitating silver particles, only to dissolved silver and suspended nanoparticles in the culture medium. No visible effects on cell morphology could be observed, independent of the chloride and FCS concentrations of the culture media (Additional file 1: Figure S4B).

Cellular uptake of nanosilver by CaCo-2 cells growing in medium A on the bottom of the culture dishes was analyzed by TEM. CaCo-2 cells exposed to nanosilver for 48 h incorporated the silver nanoparticles (Additional file 1: Figure S5). The incorporated particles were located as silver agglomerates in membrane-bound structures, most likely in endosomal–lysosomal compartments.

The release of ROS by cell cultures growing in the presence of nanosilver was also investigated. No significant amounts of ROS were produced by the CaCo-2 cells within the first 4 h of exposure to nanosilver in any of the culture media (Additional file 1: Figure S6).

In a further investigation it was shown that CaCo-2 cells growing on the bottom of the culture dishes released significant amounts of IL-8 after exposure to nanosilver (20 µg/mL). The release of IL-8 was dependent on the FCS concentration of the culture medium (Additional file 1: Figure S7A). Correlations were found between cell viability and IL-8 when CaCo-2 cells were growing in media with higher FCS concentrations in the presence of nanosilver. Floating cell cultures released less IL-8 than cells grown on the bottom of the culture dishes. The chloride concentration in the culture media had only a minor effect on the release of IL-8. However, as already mentioned for the cells growing on the bottom of the culture dishes, floating cell cultures that were growing in culture media supplemented with 10% FCS also released more IL-8 than cells growing in culture media with lower amounts of FCS (Additional file 1: Figure S7B).

## Discussion

Our results show that nanosilver toxicity was strongly affected by the composition of the culture media. Silver is known to bind strongly to inorganic and organic sulfur compounds, chloride and organic matter [32]. Thus the chloride concentration of the culture media, as well as the carbon content have an effect on silver agglomeration and complex formation, as well as on the availability of free silver ions (Ag^+^) dissolved in the culture media. In order to understand the relationships between the amount of silver, silver speciation and toxicities in vitro, we focused on the endmember media A and E (Table 2).

**Table 2:** Overview of measured parameters and observed toxicities for the endmember media A and E

	Ionic silver		Nanosilver		
	Medium A	Medium E	Medium A	Medium E	Units
Chloride	*125*	0.05	*125*	0.05	mM
Total silver in solution	0.7–1	*1–1.4*	0.6	*0.8–1.2*	µg/mL
Dissolved silver	*6–130*	0.5	*1–2.5*	0.5	ng/mL
Free silver	1.3–1.8	*3–9*	–	–	10^−10^M
Calc. free silver	0.4–9	*19*	0.06–0.2	*19–24*	10^−10^M
Toxicity floating	–	–	0	0	%
Toxicity bottom	*30–90*	10–60	*40–60*	10	%

Italics represent higher concentrations or higher toxicities

– not measured

Silver measurements in culture media taken from the upper sections of the media demonstrated that the amounts of dissolved inorganic and organic silver complexes in the culture media after the application of nanosilver or ionic silver was dependent on the chloride concentration. In culture medium E with a chloride concentration of only 0.05 mM, we observed the highest amount of total silver present, compared with the other culture media. This was true for applied nanosilver, as well as applied ionic silver. Only a very small fraction of the applied silver stayed solubilized in the cell culture system, the majority sedimented onto the cells growing on the bottom of the culture dishes. We demonstrated with the NanoSight particle tracking method that as soon as the silver nanoparticles came in contact with the culture media, agglomerates were formed. The silver agglomerates shifted within the first 24 h of incubation in cell culture media towards larger particles and then the silver agglomerates didn’t increased anymore (Additional file 1: Table S1).

The silver compounds that remained in the culture media were largely inorganic and organic silver complexes and suspended nanoparticles with a molecular weight >3 kDa. Only a very small fraction was <3 kDa, consisting of free ionic silver, and inorganic and organic silver complexes. This fraction was larger in the presence of higher chloride but the free silver ion concentration was smaller. The speciation calculations supported this finding, although much of the added dissolved silver was expected to be precipitated as AgCl, the remaining free silver concentration was lower because of greater formation of dissolved AgCl_2_ and AgCl_3_ complexes.

The amount of free ionic silver in the different culture media was therefore very low. It is known that it is mainly the free metal ion that is responsible for toxic effects and that organic and inorganic metal complexes have no or much lower toxicity [19, 21, 33–35]. This has also been observed for silver [24, 32, 36, 37]. The silver chloride complexes have different bioavailability to silver ions and are described as inducing only moderate toxicity [32, 38]. Because chloride has a higher binding constant with silver than does sulfate, replacing chloride with sulfate will increase the concentration of free ionic silver and we would thus expect to see a higher toxicity in medium E compared with medium A. However, the opposite was observed, with the highest toxicity in the high-chloride medium, which shows that the observed toxicity is not only related to the free silver concentration when free silver is present in very low concentrations.

The absence of toxic effects in the floating cell cultures shows that neither the total remaining silver in solution nor the concentration of free silver ions was enough to elicit any toxic response. The absence of toxic effects on the floating cell cultures is therefore a consequence of both the precipitation and sedimentation of dissolved silver and silver nanoparticles, as well as the complexation of the remaining silver by organic and inorganic compounds. The floating and bottom cell cultures were exposed to the same dissolved silver complexes, which were obviously non-toxic to the cells. The toxicity observed on the cells at the bottom of the culture medium was therefore not caused by dissolved silver in the bulk solution. This finding contradicts to some extent the commonly agreed mechanism of silver toxicity, which is thought to occur through dissolved silver. The total silver in solution is clearly not a good indicator of toxicity. By excluding the floating cell experiment, one might conclude that the truly dissolved silver (<3 kDa) was proportional to toxicity. However, the floating cells did not show any toxic effects.

The bottom cell cultures were of course not only exposed to silver in solution but also to the precipitated and sedimented silver particles. Local exposure of the cells was therefore mainly via silver particles covering the cells. The total silver is remaining in solution was almost the same in medium A and E (3–5% for medium A, 5–7% for medium E), so the amount sedimented onto the cells was also similar. The different toxicities in media A and E were therefore not caused by the simple presence of nanosilver on the cell surface but by the composition of the medium. The higher chloride concentration resulted in higher dissolved silver (dissolved AgCl_2_ and AgCl_3_ complexes) in the vicinity of the cell membrane. The dissolved silver concentration at the cell surface, where the concentration of total silver is very high, was therefore much greater than average over the whole solution and a locally elevated exposure concentration (LEEC) was present. LEEC has been used to explain the higher toxicity of Diuron to algae when sorbed to carbon nanotubes (CNT) than when present in the dissolved state [39]. We hypothesize that a similar mechanism was at work in the current cell culture system, that is, most of the silver in the cell culture media, when added as nanoparticles, agglomerated, precipitated and sedimented as AgCl or silver nanoparticles onto the cells at the bottom of the culture dish [40, 41]. The silver remaining in suspension or present in dissolved form had no effect on the cells. The silver on the cell surface had different bioavailability depending on the composition of the solution. Locally, the concentration of silver on the cell surface was very high and thus locally higher compared with the bulk solution. In medium A higher dissolved silver was observed in solution, so we could also expect higher dissolved silver close to the particles and therefore in the vicinity of the cell membrane.

However, there might be another mechanism not involving extracellular dissolution but rather an influence of the medium on the uptake of nanosilver and its incorporation into the cells. It has been shown that when sedimenting silver complexes come in contact with the surface of cells, a metal equilibration (adsorption–desorption) takes place [35], therefore part of the sedimented silver complex would be engulfed by the cells growing on the bottom of the culture dishes. Once inside the cells the silver complexes could dissociate and bind to intracellular proteins and have cytotoxic effects. Thus the uptake of silver complexes by the cells growing on the bottom of the culture dishes also contributed to the overall cytotoxicity of nanosilver. Similar presumptions were also reported by Gliga et al. [2] for silver nanoparticles and by Cho et al. [42] for nanogold particles.

The engulfed silver agglomerates were analyzed by scanning transmission electron microscopy and energy dispersive X-ray spectrometry. The incorporated particles were located in membrane-bound structures, most likely in endosomal-lysosomal compartments. We showed that a part of the added nanosilver particles were incorporated into the cells. We didn’t investigate the uptake of the silver nanoparticles quantitatively over time, since the focus of this investigation was the study of the influence of different chloride and FCS concentrations onto cell behavior.

Silver applied as ionic silver formed, like nanosilver, inorganic and organic complexes with compounds of the culture media, which sedimented onto the cells at the bottom of the culture dish. However, part of the applied ionic silver bound directly to proteins of the plasma membrane of the cells, when ionic silver was applied in a single batch. This ionic silver that binds directly to the proteins of the plasma membrane of the cells upon application will contribute heavily to the overall cytotoxicity of applied ionic silver (Fig. 4). The intensity of the cytotoxic effect is dependent on the amount of applied ionic silver, as well as on the types and concentrations of the inorganic and organic compounds in the medium.

## Conclusions

Silver agglomeration and silver complex formation in the culture media used in the present study were affected by chloride concentration and the presence of organic carbon (in this case, mostly FCS), and this interaction further determined the viability of the cell cultures. Cells only exposed to silver particles in suspension and dissolved silver complexes did not show any effect under all conditions. Cells growing on the bottom of the culture dishes were exposed to silver through precipitation of silver agglomerates. Dissolution of these silver compounds likely resulted in LEEC determined by the composition of the medium, and the final exposure conditions were completely different to those of a system with well-dispersed particles [40, 41]. Therefore, in addition to the required and reported material characterization, the cell culture conditions have to be carefully considered in order to estimate the type and dose of silver complexes to which cells will be exposed. These factors became more relevant when different biological systems, such as eukaryotic versus prokaryotic, are compared, which might be important, for example, when evaluating the use of silver as antibacterial coatings for implants in order to prevent bacterial colonization and biofilm formation. Silver coatings on health-care products might not only be bacteriostatic or bactericidal, but also cytotoxic. Only well-controlled and understood cultivation conditions will be able to improve the comparability of different studies using reactive nanomaterials such as silver.

## Additional file

**Additional file 1.** Additional information.

## Abbreviations

Ag^+^: silver ion
AgCl_X_^(x-1)^: silver chloride species
Ag_2_SO_4_: silver sulfate
ATCC: American type culture collection
CCMX: competence center for materials and technology
CNT: carbon nanotube
Elisa: enzyme-linked immunosorbent assay
FCS: fetal calf serum
FITC: fluorescein isothiocyanate
Genotype STR profiles: short tandem repeats genotyping
H_2_DCF-DA: 2′,7′-dichlorodihydrofluorescein-diacetate
ICP-MS: inductive coupled plasma spectroscopy-mass spectrometry
IL-8: interleukin 8
ISE: ion selective electrode
LEEC: locally elevated exposure concentration
MEM: minimum essential Eagle medium
PSN: penicillin–streptomycin–neomycin-solution
ROS: reactive oxygen species
SEM: scanning electron microscope
STEM/EDX: scanning transmission electron microscopy and energy dispersive X-ray spectrometry
TEM: transmission electron microscope
TNF-α: tumor necrosis factor α

## References

[1] Som C, Wick P, Krug H, Nowack B (2011). Environmental and health effects of nanomaterials in nanotextiles and facade coatings. Environ Int.

[2] Gliga AR, Skoglund S, Wallinder IO, Fadeel B, Karlsson HL (2014). Size-dependent cytotoxicity of silver nanoparticles in human lung cells: the role of cellular uptake, agglomeration and Ag release. Part Fibre Toxicol..

[3] Braydich-Stolle L, Hussain S, Schlager JJ, Hofmann MC (2005). In vitro cytotoxicity of nanoparticles in mammalian germline stem cells. Toxicol Sci.

[4] Jo HJ, Choi JW, Lee SH, Hong SW (2012). Acute toxicity of Ag and CuO nanoparticle suspensions against Daphnia magna: the importance of their dissolved fraction varying with preparation methods. J Hazard Mater.

[5] Johnston HJ, Hutchison G, Christensen FM, Peters S, Hankin S, Stone V (2010). A review of the in vivo and in vitro toxicity of silver and gold particulates: particle attributes and biological mechanisms responsible for the observed toxicity. Crit Rev Toxicol.

[6] Beer C, Foldbjerg R, Hayashi Y, Sutherland DS, Autrup H (2012). Toxicity of silver nanoparticles–nanoparticle or silver ion?. Toxicol Lett.

[7] Wijnhoven SWP, Peijnenburg WJGM, Herberts CA, Hagens WI, Oomen AG, Heugens EHW, Roszek B, Bisschops J, Gosens I, Van de Meent D (2009). Nano-silver—a review of available data and knowledge gaps in human and environmental risk assessment. Nanotoxicology..

[8] Martirosyan A, Bazes A, Schneider YJ (2014). In vitro toxicity assessment of silver nanoparticles in the presence of phenolic compounds—preventive agents against the harmful effect?. Nanotoxicology..

[9] Foldbjerg R, Olesen P, Hougaard M, Dang DA, Hoffmann HJ, Autrup H (2009). PVP-coated silver nanoparticles and silver ions induce reactive oxygen species, apoptosis and necrosis in THP-1 monocytes. Toxicol Lett.

[10] Pratsinis A, Hervella P, Leroux JC, Pratsinis SE, Sotiriou GA (2013). Toxicity of silver nanoparticles in macrophages. Small.

[11] Xiu ZM, Zhang QB, Puppala HL, Colvin VL, Alvarez PJ (2012). Negligible particle-specific antibacterial activity of silver nanoparticles. Nano Lett.

[12] Kim S, Choi JE, Choi J, Chung KH, Park K, Yi J, Ryu DY (2009). Oxidative stress-dependent toxicity of silver nanoparticles in human hepatoma cells. Toxicol In Vitro.

[13] Singh RP, Ramarao P (2012). Cellular uptake, intracellular trafficking and cytotoxicity of silver nanoparticles. Toxicol Lett.

[14] Greulich C, Braun D, Peetsch A, Diendorf J, Siebers B, Epple M, Koller M (2012). The toxic effect of silver ions and silver nanoparticles towards bacteria and human cells occurs in the same concentration range. Rsc Adv.

[15] Hoheisel SM, Diamond S, Mount D (2012). Comparison of nanosilver and ionic silver toxicity in *Daphnia magna* and *Pimephales promelas*. Environ Toxicol Chem.

[16] Behra R, Sigg L, Clift MJ, Herzog F, Minghetti M, Johnston B, Petri-Fink A, Rothen-Rutishauser B (2013). Bioavailability of silver nanoparticles and ions: from a chemical and biochemical perspective. J R Soc Interface.

[17] Handy RD, Owen R, Valsami-Jones E (2008). The ecotoxicology of nanoparticles and nanomaterials: current status, knowledge gaps, challenges, and future needs. Ecotoxicology.

[18] Hinderliter PM, Minard KR, Orr G, Chrisler WB, Thrall BD, Pounds JG, Teeguarden JGISDD (2010). A computational model of particle sedimentation, diffusion and target cell dosimetry for in vitro toxicity studies. Part Fibre Toxicol..

[19] Anderson BS, Hunt JW, Piekarski WJ, Phillips BM, Englund MA, Tjeerdema RS, Goetzl JD (1995). Influence of salinity on copper and azide toxicity to larval topsmelt *Atherinops affinis* (Ayres). Arch Environ Contam Toxicol.

[20] Kakinen A, Bondarenko O, Ivask A, Kahru A (2011). The effect of composition of different ecotoxicological test media on free and bioavailable copper from CuSO4 and CuO nanoparticles: comparative evidence from a Cu-selective electrode and a Cu-biosensor. Sensors (Basel)..

[21] Park S, Woodhall J, Ma G, Veinot JG, Cresser MS, Boxall AB (2013). Regulatory ecotoxicity testing of engineered nanoparticles: are the results relevant to the natural environment?. Nanotoxicology..

[22] Chambers BA, Afrooz AR, Bae S, Aich N, Katz L, Saleh NB, Kirisits MJ (2014). Effects of chloride and ionic strength on physical morphology, dissolution, and bacterial toxicity of silver nanoparticles. Environ Sci Technol.

[23] Chernousova S, Epple M (2013). Silver as antibacterial agent. Ion, nanoparticle, and metal. Angew Chem Int Ed Engl.

[24] Ahlberg S, Antonopulos A, Diendorf J, Dringen R, Epple M, Flock R, Goedecke W, Graf C, Haberl N, Helmlinger J (2014). PVP-coated, negatively charged silver nanoparticles: a multi-center study of their physicochemical characteristics, cell culture and in vivo experiments. Beilstein J Nanotechnol..

[25] Loza KJD, Sengstock C, Ruiz-Gonzales L, Gonzales-Calbet JM, Vallet-Regi M, Köller M, Epple M (2014). The dissolution and biological effects of silver nanoparticles in biological media. J Mater Chem B..

[26] Kaiser JP, Roesslein M, Diener L, Wick P (2013). Human health risk of ingested nanoparticles that are added as multifunctional agents to paints: an in vitro study. PLoS ONE.

[27] Smulders S, Kaiser JP, Zuin S, Van Landuyt KL, Golanski L, Vanoirbeek J, Wick P, Hoet PH (2012). Contamination of nanoparticles by endotoxin: evaluation of different test methods. Part Fibre Toxicol..

[28] Hole P, Sillence K, Hannell C, Maguire CM, Roesslein M, Suarez G, Capracotta S, Magdolenova Z, Horev-Azaria L, Dybowska A (2013). Interlaboratory comparison of size measurements on nanoparticles using nanoparticle tracking analysis (NTA). J Nanopart Res.

[29] Gustafsson JP. Visual Minteq ver. 3.0; KTH. Stockholm; 2012.

[30] Thurnherr T, Brandenberger C, Fischer K, Diener L, Manser P, Maeder-Althaus X, Kaiser JP, Krug HF, Rothen-Rutishauser B, Wick P (2011). A comparison of acute and long-term effects of industrial multiwalled carbon nanotubes on human lung and immune cells in vitro. Toxicol Lett.

[31] Kaiser JP, Buerki-Thurnherr T, Wick P (2013). Influence of single walled carbon nanotubes at subtoxical concentrations on cell adhesion and other cell parameters of human epithelial cells. J King Saud Univ—Sci.

[32] Levard C, Hotze EM, Lowry GV, Brown GE (2012). Environmental transformations of silver nanoparticles: impact on stability and toxicity. Environ Sci Technol.

[33] Erickson RJ, Benoit DA, Mattson VR, Nelson HP, Leonard EN (1996). The effects of water chemistry on the toxicity of copper to fathead minnows. Environ Toxicol Chem.

[34] Hsiao IL, Huang YJ (2013). Effects of serum on cytotoxicity of nano-and micro-sized ZnO particles. J Nanopart Res.

[35] Zhao C-M, Campbell PGP, Wilkinson KJ (2016). When are metal complexes bioavailable?. Environ Chem.

[36] Ratte HT (1999). Bioaccumulation and toxicity of silver compounds. A review. Environ Sci Technol.

[37] Schierholz JM, Wachol-Drewek Z, Lucas LJ, Pulverer G (1998). Activity of silver ions in different media. Zentralbl Bakteriol..

[38] Hogstrand C, Galvez F, Wood CM (1996). Toxicity, silver accumulation and metallothionein induction in freshwater rainbow trout during exposure to different silver salts. Environ Toxicol Chem.

[39] Schwab F, Bucheli TD, Camenzuli L, Magrez A, Knauer K, Sigg L, Nowack B (2013). Diuron sorbed to carbon nanotubes exhibits enhanced toxicity to Chlorella vulgaris. Environ Sci Technol.

[40] Wittmaack K (2011). Excessive delivery of nanostructured matter to submersed cells caused by rapid gravitational settling. ACS Nano.

[41] Wittmaack K (2011). Novel dose metric for apparent cytotoxicity effects generated by in vitro cell exposure to silica nanoparticles. Chem Res Toxicol.

[42] Cho EC, Zhang Q, Xia Y (2011). The effect of sedimentation and diffusion on cellular uptake of gold nanoparticles. Nat Nanotechnol.

