# Supplementary material for: Cytotoxic effects of nanosilver are highly dependent on the chloride concentration and the presence of organic compounds in the cell culture media
Source: J Nanobiotechnology. 2017 Jan 6;15:5. doi: 10.1186/s12951-016-0244-3 (PMC5219688; doi:10.1186/s12951-016-0244-3)
Supplement: Supplementary file 1 — Additional file 1. Additional information. [file 12951_2016_244_MOESM1_ESM.docx]

Cytotoxic Effects of Nanosilver are Highly Dependent on the Chloride Concentration and the Presence of Organic Compounds in the Cell Culture Media

Jean-Pierre Kaiser,^a^* Matthias Roesslein,^a^ Liliane Diener,^a^ Adrian Wichser,^a^ Bernd Nowack^b^ and Peter Wick^a^

^a^Empa, Swiss Federal Laboratories for Material Science and Technology, Materials meet Life, Particles-Biology Interactions Laboratory, Lerchenfeldstrasse 5, 9014 St. Gallen, Switzerland

^b^Empa, Swiss Federal Laboratories for Materials Science and Technology, Technology and Society Laboratory, Lerchenfeldstrasse 5, CH-9014 St. Gallen, Switzerland

**Supporting Information**

**Table S1** Agglomeration behavior of nanosilver during incubation in different culture media

| Culture  medium | Size  maxima at 0 h | Size  distribution range at  0 h | Size  maxima  after 24 h | Size  distribution range after 24 h | Size  maxima  after 48 h | Size  distribution range after 48 h | Size  maxima  after 72 h | Size  distribution range after 72 h |
| --- | --- | --- | --- | --- | --- | --- | --- | --- |
| A | 129 nm | 94 nm - 212 nm | 163 nm | 109 nm - 237 nm | 158 nm | 100 nm - 237 nm | 146 nm | 67 nm - 216 nm |
| B | 136 nm | 84 nm - 201 nm | 147 nm | 102 nm - 221 nm | 139 nm | 81 nm - 219 nm | 149 nm | 94 nm - 229 nm |
| C | 134 nm | 74 nm - 231 nm | 133 nm | 83 nm - 211 nm | 150 nm | 80 nm - 214 nm | 141 nm | 103 nm - 231 nm |
| D | 133 nm | 63 nm - 194 nm | 147 nm | 103 nm - 218 nm | 137 nm | 80 nm - 214 nm | 141 nm | 94 nm - 217 nm |
| E | 141 nm | 83 nm - 214 nm | 143 nm | 65 nm - 203 nm | 135 nm | 79 nm - 211 nm | 142 nm | 102 nm - 222 nm |

**Table S1** Agglomeration behavior of nanosilver was analyzed by a particle tracking method (NanoSight LM20). Nanosilver particles (100 µg/mL) were incubated in culture media with 10% fetal calf serum at 37°C for 0 h, 24 h, 48 h and 72 h.

Standard deviation: ± 5 nm

**Figure S1** CaCo-2 cells grown in the presence of 20 µg/mL nanosilver


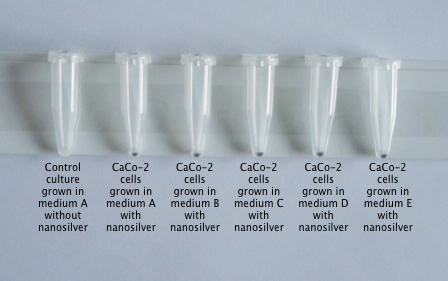


CaCo-2 cells grown in culture media with different chloride concentrations (media A - E) in the presence of 20 µg/mL nanosilver after an incubation period of 48 h.

CaCo-2 cells growing on the bottom of the culture dishes were exposed to precipitating silver agglomerates and silver complexes (shown in tubes), which adsorbed onto the extracellular membrane of the cells.

**Figure S2** CaCo-2 cells grown in culture medium A in the presence of 150 µg/mL nanosilver


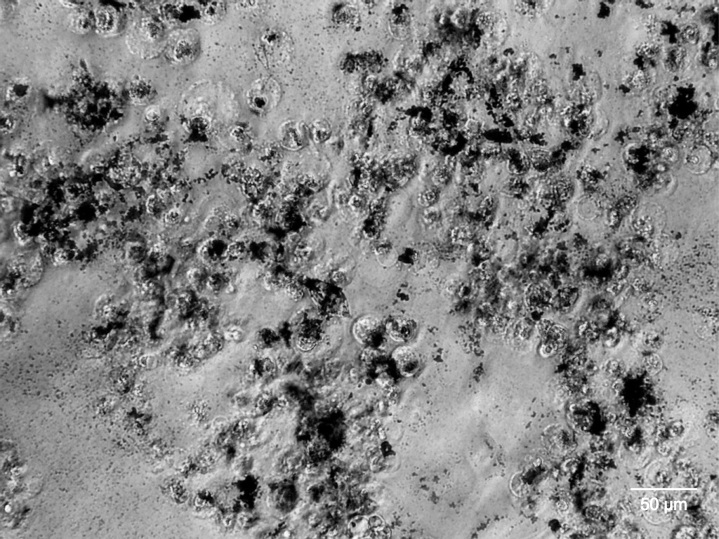


CaCo-2 cells grown in culture medium A with physiological chloride concentration and 10 % fetal calf serum in the presence of 150 µg/mL nanosilver after an incubation period of 48 h. Larger silver agglomerates adsorbed onto the extracellular membrane of the cells.

**Figure S3** Viability of CaCo-2 cell cultures grown in culture media in the absence of nanosilver


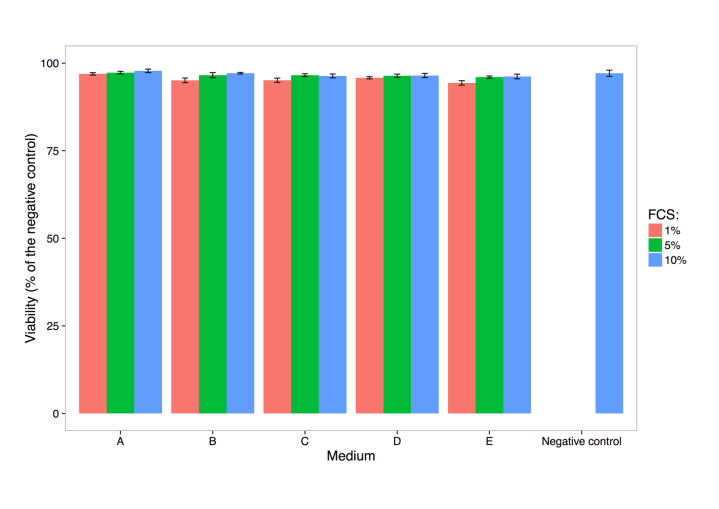


Viability of CaCo-2 cell cultures grown in culture media with different chloride concentrations (media A - E) in the absence of nanosilver for 48 h. Viability of cells grown on the bottom of the culture dishes were not affected when the sodium chloride in the culture medium was replaced by sodium sulfate.

The negative control culture corresponded to media E with 10 % fetal calf serum (FCS).

**Figure S4** Cell morphology of CaCo-2 cell cultures grown in culture media supplemented with nanosilver


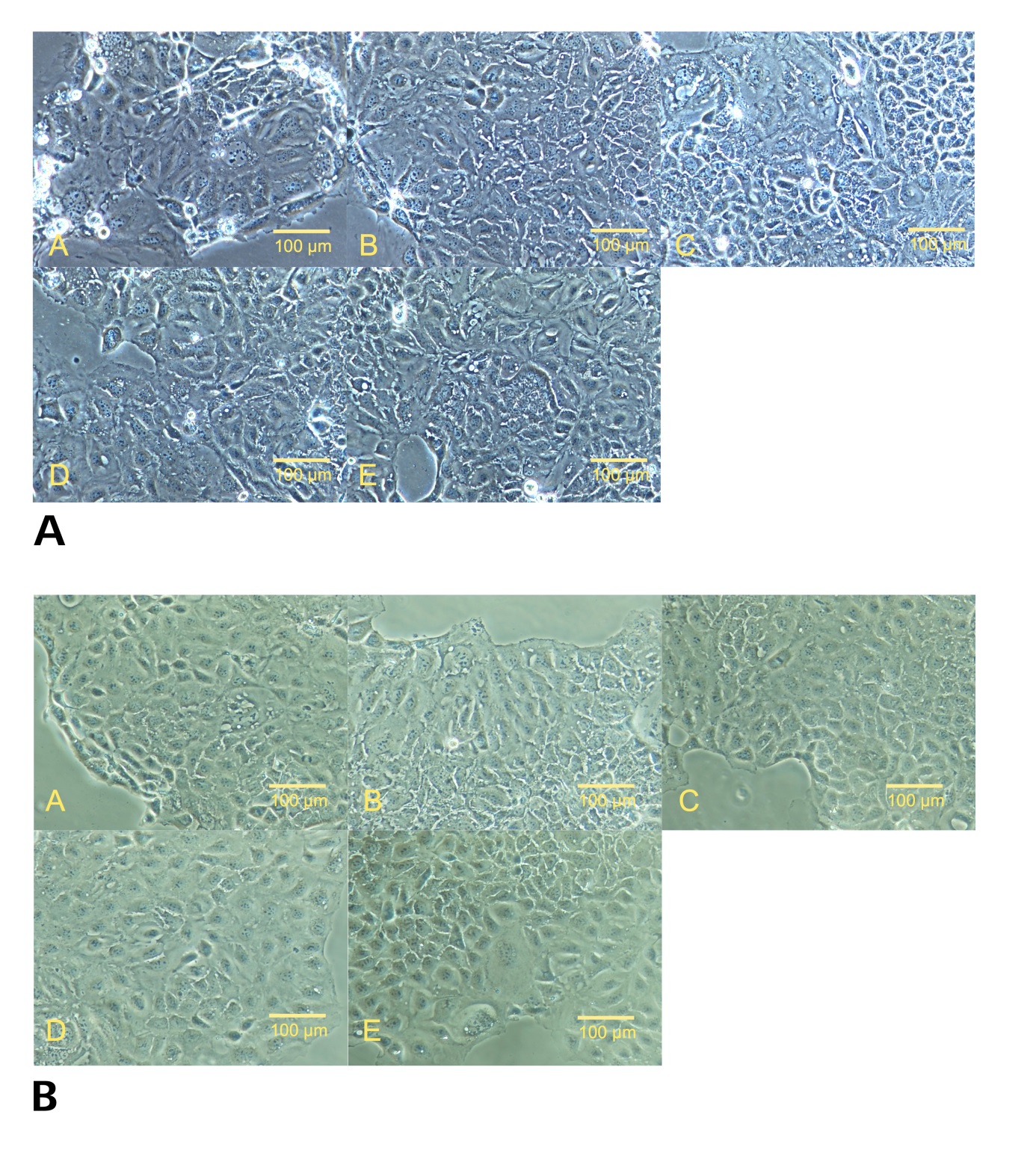


Cell morphology of cultures grown in culture media with different chloride concentrations (media A - E as labeled) in the presence of 20 µg/mL nanosilver and 10 % fetal calf serum for 48 h (A) bottom cell cultures and (B) floating cell cultures. Nanosilver affected the morphology of the bottom cell cultures. The morphology of cell clusters was mostly affected when the cells were growing in culture media A.

**Figure S5** Engulfment of nanosilver by CaCo-2 cells exposed to nanosilver


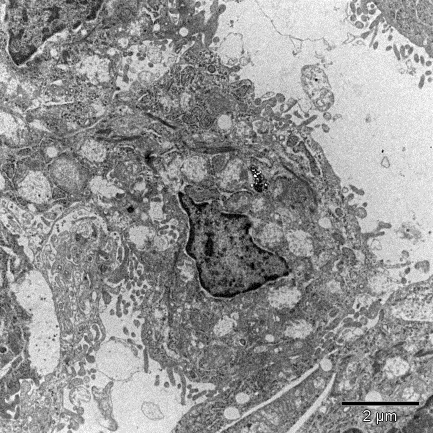


*


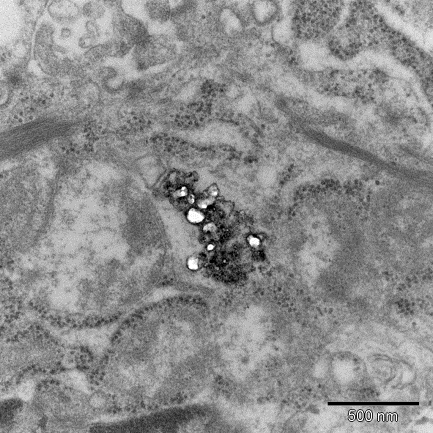


*

Engulfment of nanosilver by CaCo-2 cells exposed to nanosilver (20 µg/mL) for 48 h. A higher amount of nanosilver is incorporated into the cells.

* Nanosilver agglomerates

**Figure S6** Release of reactive oxygen species (ROS) by gastrointestinal cells (CaCo-2)


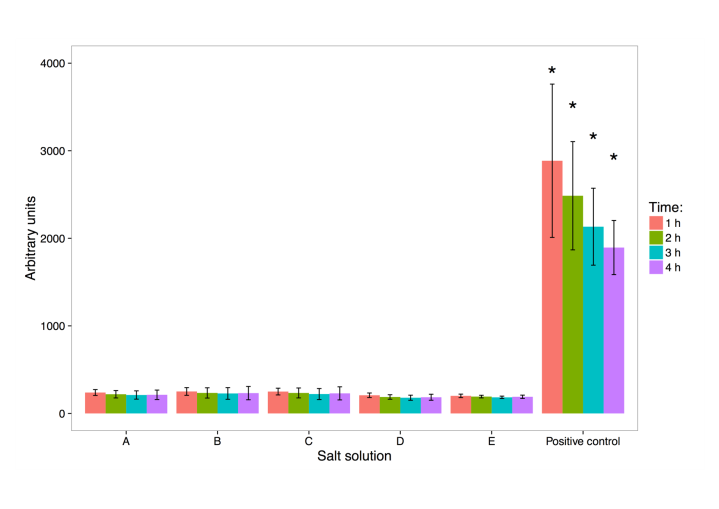


The release of ROS was analyzed after an exposure time of 1 h, 2 h, 3 h, 4 h. 20 µg/mL nanosilver were dissolved in salt solutions with different chloride concentrations. The chloride concentrations in the salt solutions corresponded to the chloride and sulfate concentrations in the media A - E. The salt solutions contained only the cations and anions of the corresponding culture media without fetal calf serum, penicillin-streptomycin-neomycin-solution, glutamine solution, non-essential amino acid solution, sodium pyruvate solution, vitamin solution and phenol red. The different chloride concentrations in the salt solutions did not induce ROS.

Salt solutions with different chloride concentrations without nanosilver were used as negative controls.

3-Morpholinosydnonimine hydrochloride (1 mM) was used as positive control.

* Significantly different from the negative control

**Figure S7** Cytokine release by CaCo-2 cell cultures


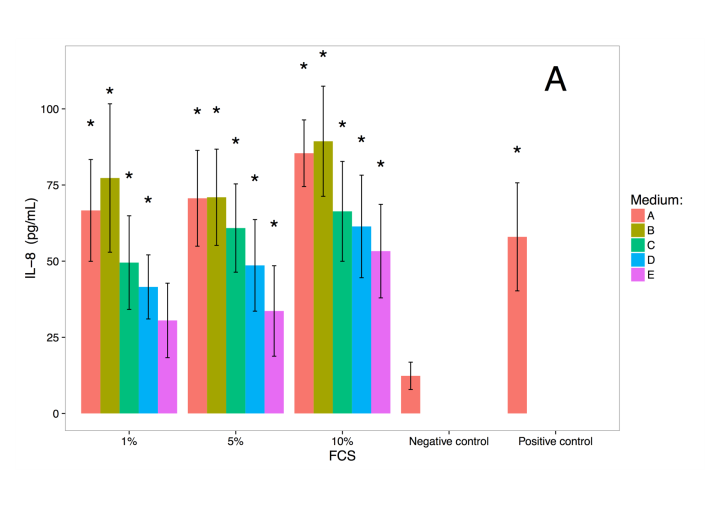

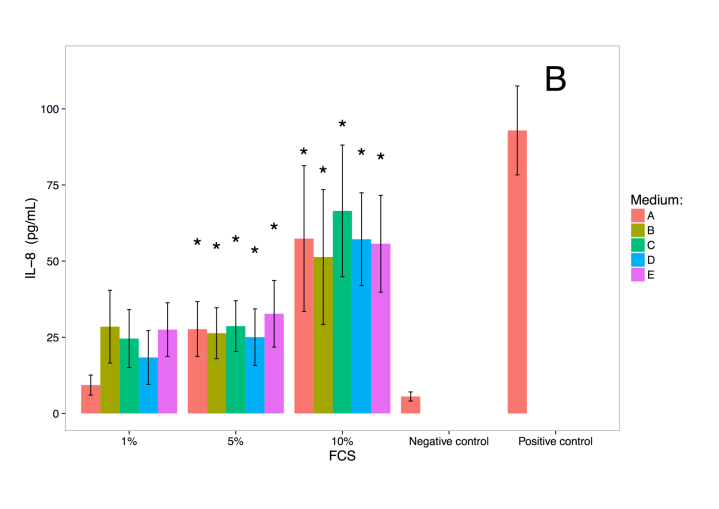


Release of interleukin 8 (IL-8) in culture media with different chloride concentrations (media A - E) in the presence of 20 µg/mL nanosilver: (A) bottom cell cultures and (B) floating cell cultures. Higher amounts of IL-8 were released in culture media with physiological chloride concentrations.

Negative control: No nanosilver

Positive control: TNF-α (50 ng/mL)

* Significantly different from the negative control
